# Editorial: Immunological challenges around pregnancy complications associated with failures of maternal tolerance to the fetus

**DOI:** 10.3389/fimmu.2022.983739

**Published:** 2022-08-01

**Authors:** Michael Eikmans, Diana M. Morales-Prieto, Marie-Louise van der Hoorn, Udo R. Markert

**Affiliations:** ^1^ Leiden University Medical Center, Leiden, Netherlands; ^2^ Placenta Lab, Department of Obstetrics, Jena University Hospital, Jena, Germany

**Keywords:** pregnancy, trophoblast, immune cells, preeclampsia, recurrent pregnancy loss, chronic histiocytic intervillositis

Successful pregnancy provides an intriguing immunologic paradox. The fetus carries both paternal and maternal genes and therefore is 50% foreign to the mother, but is tolerized by the maternal immune system. Several immune regulatory mechanisms are at play at the maternal-fetal interface, which represents the location where fetal trophoblasts in the placenta come into contact with maternal immune cells. In early pregnancy, extravillous trophoblasts are relevant in plugging and remodeling maternal spiral arteries and thereby are relevant for the proper development of the placenta and the fetus. At the same time, fetal trophoblasts at the maternal-fetal interface need to be tolerized against immune attack by maternal T cells, myeloid cells (macrophages and dendritic cells), and decidual natural killer (NK) cells. One way of establishing this is the expression of HLA-G on the surface of trophoblasts and the secretion of soluble HLA-G. A second way is the generation and development of regulatory immune cells, including regulatory T cells (Tregs) and myeloid-derived suppressor cells (MDSCs). These cell types mediate homeostasis by dampening effector immune cell mechanisms.

This Research Topic focuses on mechanisms that effectuate a tolerogenic microenvironment in healthy pregnancy and describes how these features may be disturbed in various types of pregnancy complications.

An experimental study by Dietz et al. uses a murine model lacking the Qa-2 antigen, a murine homologue-candidate for HLA-G, to show the effect of sHLA-G in reducing abortion rates through inducing Tregs and MDSCs. Both cell types are shown to suppress the action of effector T cells. Interaction and cross-talk between the trophoblast and macrophage may lead to direct polarization of the latter, as reviewed by Ding et al.. Such effects mediating tolerogenicity can be driven either by direct cell-cell contact, excreted soluble factors or a combination of the two. Next to macrophages, dendritic cells make part of the myeloid cell lineage. These cells are the most efficient type of antigen-presenting cells, and as part of the decidual environment, they are thought to drive fetus-specific immunity by the maternal immune system. Zhang et al. show that the female steroid hormone progesterone has major effects on the characteristics of dendritic cells. Another regulatory mechanism at the maternal-fetal interface may be directed by surface molecules CD39 and CD73, which promote conversion of adenosine 5′-triphosphate (ATP) to adenosine and thereby change a pro-inflammatory to an anti-inflammatory environment. Zhu et al. demonstrate that trophoblasts express both molecules and also show that a high fraction of decidual NK cells are positive for CD39. Expression of both molecules was found to be decreased at the maternal-fetal interface in women with pregnancy loss, thereby emphasizing their importance in this disease.

The current Research Topic is further directed at pregnancy-related complications with possible involvement of the immune system, which are studied here. These include recurrent pregnancy loss (RPL), chronic histiocytic intervillositis (CHI), and preeclampsia.

As the fetal cells are tolerized by the maternal immune system in healthy pregnancy, immunological mechanisms and disturbances therein may underlie RPL. Women with RPL have had two or more spontaneous miscarriages. It affects approximately 3% of all fertile couples and in more than 50% of couples the underlying cause is not clear: a considerable part of such cases are suspected to have an immunologic nature. Li et al. outline how big data analysis of the maternal-fetal interface, as well as placenta and blood, from women with RPL may help elucidating the pathogenesis.

CHI is a poorly understood histopathological lesion of the placenta. CHI is significantly associated with miscarriage, fetal growth restriction, and intrauterine fetal death. In this disease, an intervillous infiltrate, which is of maternal origin, can be seen in every trimester throughout gestation. The interplay between maternal immune cells and fetal trophoblasts in CHI might point toward a breach in immune tolerance and is a topic for ongoing research. Cornish et al. present a detailed overview of prevalence, pathology, and clinical consequences of different gestational syndromes, including CHI, which are associated with adverse pregnancy outcomes. Brady et al. describe how application of immune modulatory medication in women with CHI may improve placental lesions and higher the chance of livebirth.

The third type of pregnancy-related complication discussed in this Research Topic is preeclampsia, which is a serious complication endangering the mother and the fetus. High blood pressure is an important indicator for diagnosing preeclampsia. Gan et al. found that systolic blood pressure of the mother at 12 weeks of gestation predicts the development of severe preeclampsia and of low birth weight of the baby. Green et al. present a systematic review concerning the role of Tregs in adverse pregnancy outcome. They show that decreased Treg numbers in the peripheral blood of pregnant women is significantly associated with the occurrence of pre-eclampsia. Acute atherosis is a lesion which can be present at the maternal spiral arteries, and which may be encountered in preeclampsia. Jacobsen et al. provide an extensive overview and discussion on acute atherosis, including its definition, underlying mechanism, and possible clinical consequences.

In summary, this Research Topic provides insights in the immunological challenges around pregnancy complications associated with failures of maternal tolerance to the fetus. The collection of articles highlights the function of specific immune cell populations and describes new mechanisms of pregnancy monitoring that can contribute to advanced basic research and clinical translation for various types of pregnancy complications.

## Author contributions

ME, M-LH, DM-P, and UM composed the text, revised it, and approved the final draft.

## Conflict of interest

The authors declare that the research was conducted in the absence of any commercial or financial relationships that could be construed as a potential conflict of interest.

## Publisher’s note

All claims expressed in this article are solely those of the authors and do not necessarily represent those of their affiliated organizations, or those of the publisher, the editors and the reviewers. Any product that may be evaluated in this article, or claim that may be made by its manufacturer, is not guaranteed or endorsed by the publisher.

